# An Exploratory One Health Investigation of *Mycobacterium tuberculosis* Complex at the Camel–Human Interface in Abu Dhabi Emirate, United Arab Emirates

**DOI:** 10.1155/tbed/7098203

**Published:** 2026-09-21

**Authors:** Gobena Ameni, Aboma Zewude, Asha Antony, Tsegaye Shamebo, Temesgen Mohammed, Nabeeha Hassan Abdelgaleel, Mohamud Sheek-Hussein, Balázs Ádám, Robert Barigye, Markos Tibbo

**Affiliations:** ^1^ Department of Veterinary Medicine, College of Agriculture and Veterinary Medicine, United Arab Emirates University, Al Ain, UAE, uaeu.ac.ae; ^2^ Department of Health Sciences, College of Natural and Health Sciences, Zayed University, Dubai, UAE, zu.ac.ae; ^3^ Institute of Public Health, College of Medicine and Health Sciences, United Arab Emirates University, Al Ain, Abu Dhabi Emirate, UAE, uaeu.ac.ae; ^4^ School of Public Health, Loma Linda University, Loma Linda, California, USA, llu.edu; ^5^ Subregional Office for the Gulf Cooperation Council States and Yemen, Food and Agriculture Organization of the United Nations, Abu Dhabi, UAE, fao.org

**Keywords:** abattoir workers, dromedary camel, LIODetect TB-ST, *Mycobacterium tuberculosis* complex, One Health, seroreactivity

## Abstract

This exploratory cross‐sectional One Health study characterized LIODetect TB‐ST antibody reactivity in dromedary camels and abattoir workers in Abu Dhabi Emirate and investigated tuberculosis‐compatible lesions in slaughtered camels. Sera from 356 camels sampled from farms and livestock holdings (izbas), 393 camels presented for slaughter at Al Bawadi Abattoir, and 86 abattoir workers were screened with LIODetect TB‐ST. All 25 LIODetect‐reactive slaughtered camels underwent detailed postmortem examination; nonreactive slaughtered camels were not examined postmortem. Lesions identified in two reactive camels were evaluated by histopathology, Ziehl–Neelsen staining, and IS6110 polymerase chain reaction (PCR). LIODetect reactivity was detected in 33/356 field camels (9.3%, 95% CI: 6.5%–12.8%), 25/393 slaughtered camels (6.4%, 95% CI: 4.2%–9.2%), and 3/86 workers (3.5%, 95% CI: 0.7%–9.9%). No recorded characteristic was associated with reactivity among field camels. Among slaughtered camels, age group and body condition showed exploratory statistical associations with reactivity, but category‐specific estimates were imprecise. Two of the 25 reactive slaughtered camels (8.0%) had granulomatous lymphadenitis with acid‐fast bacilli, and lesion tissue from both animals contained IS6110‐detectable *Mycobacterium tuberculosis* complex (MTBC) DNA. LIODetect TB‐ST has not been validated for prevalence estimation in dromedaries; therefore, these proportions describe assay reactivity rather than true infection prevalence. Because postmortem examination was restricted to reactive animals, the 2/25 finding cannot estimate lesion prevalence, false‐negative frequency, or positive predictive value. The three reactive workers represent an occupational‐health surveillance signal, not evidence of active tuberculosis or camel‐to‐human transmission. Systematic meat inspection, validated camel diagnostics, culture and species‐level molecular characterization, traceable animal movements, and linked veterinary and occupational‐health surveillance are needed.

## 1. Introduction

Tuberculosis caused by members of the *Mycobacterium tuberculosis* complex (MTBC) affects humans and a wide range of domestic and wild animals. The complex includes host‐adapted lineages such as *M. tuberculosis*, *M*. *africanum*, *M. bovis*, and *M. caprae*, although host specificity is incomplete and cross‐species infection occurs [1, 2]. Animal tuberculosis can reduce productivity, cause carcass or organ condemnation, restrict animal movement and trade, and expose people through direct contact, aerosols, or consumption of contaminated unpasteurized products [3, 4]. These linked consequences place MTBC surveillance within a One Health framework.

Dromedary camels (*Camelus dromedarius*) are biologically susceptible to several MTBC members, including *M. bovis*, *M. tuberculosis*, and *M. caprae*, although the frequency and epidemiological importance of each species vary by setting [5–8]. Reports from Africa, the Middle East, and Asia have described tuberculous lesions and microbiologically confirmed infection in dromedaries. In the United Arab Emirates (UAE), an outbreak of *M. bovis* in a racing herd showed that clinically important camel tuberculosis can occur and that antibody assays may contribute to herd investigation when interpreted alongside pathology and direct detection [9].

Camels have substantial food security contributions, economic, and cultural importance in the UAE. The Abu Dhabi Agriculture and Food Safety Authority reported ~476,000 camels in Abu Dhabi Emirate in 2024, distributed across the Abu Dhabi, Al Ain, and Al Dhafra regions [10]. Production systems range from traditional livestock holdings (izbas) to commercial dairy and breeding enterprises. Animal movements, livestock markets, intensification, and close contact among camels, owners, veterinarians, traders, and abattoir personnel create opportunities for exposure to infectious agents in both animal‐to‐human and human‐to‐animal directions. Published information on camel‐associated MTBC in the UAE nevertheless remains sparse, while national human surveillance data cannot identify zoonotic or reverse‐zoonotic transmission pathways without species‐ and strain‐level microbiology [11].

Serological assays are operationally attractive for field screening, but antibody detection does not distinguish active disease from previous exposure or resolved infection, and diagnostic performance is host‐ and context‐dependent. No study has established validated sensitivity, specificity, likelihood ratios, or predictive values for LIODetect TB‐ST specifically in dromedary camels. Evidence from dromedaries supports the general principle of antibody‐based screening but does not validate the assay used here. In a 58‐animal racing herd in Dubai, a different Chembio lateral‐flow rapid test and a multiantigen print immunoassay detected antibodies in all three dromedaries with culture‐confirmed *M. bovis* infection, while the remaining 55 animals were nonreactive; however, only seroreactive animals underwent postmortem confirmation, and the authors explicitly called for larger validation studies [9]. Other camel studies have likewise used lateral‐flow antibody tests with limited or selectively verified reference standards [12, 13]. In cattle, Zewude et al. [14] reported 54% sensitivity and 98.8% specificity for the LIONEX Animal TB rapid test against a combined single intradermal comparative tuberculin and interferon gamma test (SICTT/IFN‐γ) as a reference test, with substantial agreement with SICTT (*κ* = 0.63). In humans, La Manna et al. [15] reported 73.3% sensitivity and 97.4% specificity for LIODetect TB‐ST. These estimates cannot be extrapolated directly to dromedaries. Current WOAH guidance recommends the use of serological assays in camels since the cell‐mediated tests have not been optimized in camels so far [16].

This study assessed LIODetect TB‐ST seroreactivity in camels kept on farms and izbas across the Abu Dhabi Emirate, camels presented for slaughter at Al Bawadi Abattoir in Al Ain, and workers employed at that abattoir. All 25 LIODetect‐reactive slaughtered camels underwent detailed postmortem examination; nonreactive slaughtered camels were not examined postmortem. Lesions identified in two reactive camels were evaluated by histopathology, acid‐fast staining, and IS6110 polymerase chain reaction (PCR). To our knowledge, this is the first UAE study to examine these three populations concurrently while linking slaughter‐camel antibody screening to pathological and MTBC‐targeted molecular investigation. The study was designed as an exploratory surveillance assessment, not to establish true prevalence, diagnostic accuracy, or camel‐to‐human transmission.

## 2. Materials and Methods

### 2.1. Study Design and Setting

An exploratory cross‐sectional study was conducted in Abu Dhabi Emirate, encompassing the Abu Dhabi, Eastern (Al Ain), and Al Dhafra regions. The field‐camel component was sampled from May 2022 to March 2023 as part of a broader One Health camel‐health survey [17], while the Al Bawadi Abattoir camel and worker component was sampled between March 2022 and July 2023 [18]. These periods spanned cool and hot seasons, but season was not retained as an analytical variable in the MTBC dataset, and no seasonal effect was estimated. Camels were sampled from commercial farms and two locally recorded categories of izba. In Abu Dhabi regulatory usage, an izba is a livestock holding used for keeping animals such as camels, sheep, and goats rather than a village or hamlet [19, 20]. In the study records, a regular izba denoted a formally allocated or recognized livestock holding within the standard regulatory framework, whereas a random (irregular) izba denoted an informally established holding outside that framework. These categories describe the administrative status and do not imply specific herd size, production intensity, or biosecurity performance. Al Bawadi Abattoir was selected because it receives camels from livestock markets and multiple source areas and therefore provides a practical convergence point for animal inspection and occupational exposure. Individual movement and country‐of‐origin records for the slaughtered camels were incomplete.

**Figure 1:**
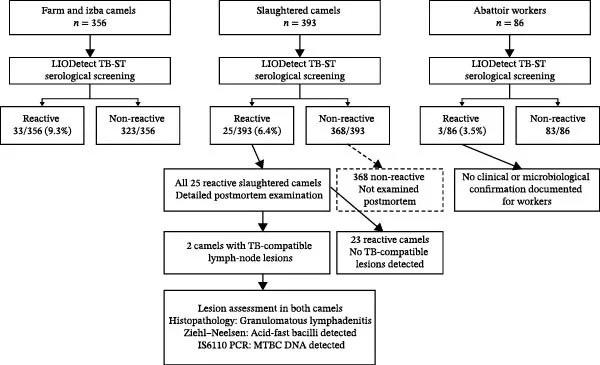
Study sampling and diagnostic verification pathway. The three sampling streams comprised 356 field camels from farms and izbas, 393 slaughtered camels, and 86 abattoir workers. All were screened with LIODetect TB‐ST. Detailed postmortem examination was restricted to the 25 LIODetect‐reactive slaughtered camels; the 368 nonreactive slaughtered camels were not examined postmortem. Two reactive camels had TB‐compatible lymph‐node lesions; histopathology showed granulomatous lymphadenitis, Ziehl–Neelsen staining demonstrated acid‐fast bacilli, and IS6110 PCR detected MTBC DNA in lesion material. No clinical or microbiological confirmation was documented for the workers. The selective postmortem pathway is a source of partial verification bias. Reactive = visible control band plus ≥1 visible test band. LIODetect antibody reactivity alone does not establish active tuberculosis.

The study comprised three sampling streams: field camels from farms and izbas, camels presented for slaughter at the Al Bawadi Abattoir, and workers employed at that abattoir. Animal and worker data were analyzed separately because no paired camel–worker exposure units, longitudinal follow‐up, or molecular strain comparisons were available. The study structure and verification pathway are summarized in Figure 1.

### 2.2. Study Populations, Sample Size, and Recorded Variables

#### 2.2.1. Camels From Farms and Izbas

The original protocol calculated a simple‐random‐sample target of ~400 camels using 50% expected prevalence, 5% absolute precision, and 95% confidence and proposed a cluster‐adjusted target of 1200. Operational field sampling yielded 356 camels from 102 holdings. In the parent survey, holdings were randomly selected from the Abu Dhabi Agriculture and Food Safety Authority registry after stratification by region, and camels were randomly sampled within the selected holdings [17]. The 102 holdings comprised 31 in the Abu Dhabi Region, 52 in the Eastern Region, and 19 in Al Dhafra; administratively, they comprised 11 farms, 59 regular izbas, and 32 random izbas [17]. For the MTBC analysis, the retained animal‐level dataset contained the region and holding type but not a unique holding identifier or the exact number of sampled camels per holding. Consequently, within‐holding clustering could not be modeled, and the analysis should be interpreted as exploratory rather than as a design‐based population estimate.

The recorded animal‐level variables were region, holding type, age class, body condition, and origin. Age was classified in field records as young or adult; body condition as poor, medium, or good; and origin as born in the UAE, introduced, or unknown. No observations were missing for region, holding type, age class, or body condition; origin was unknown for one camel.

#### 2.2.2. Slaughtered Camels

Blood samples were obtained from 393 camels presented for slaughter at Al Bawadi Abattoir during the abattoir sampling period [18]. The recorded variables were sex, age group (<4, 4–7, or >7 years), and body condition (poor, medium, or good); these variables were complete for all 393 animals. All 25 LIODetect‐reactive slaughtered camels underwent detailed postmortem examination for lesions compatible with tuberculosis. Nonreactive slaughtered camels were not examined postmortem. Reliable individual animal‐movement and country‐of‐origin data were unavailable.

#### 2.2.3. Abattoir Workers

Eighty‐six of ~110 workers at Al Bawadi Abattoir consented to participate in the parent occupational component [17, 18]. All participants were male. The recorded variables available for the present analysis were age group (≤30, >30–40, or >40 years), job category (butcher or other), duration of employment (≤5, >5–10, or >10 years), and nationality group; no observations were missing for these variables. Detailed measures of contact intensity, use of personal protective equipment, previous tuberculosis history, BCG vaccination, household exposure, and nonoccupational risk factors were not available in the retained dataset. The serological survey did not include clinical diagnosis, chest imaging, interferon‐γ release assays, sputum testing, mycobacterial culture, or molecular linkage to camel isolates. Clinical referral and confirmatory follow‐up were not documented in the available study records.

### 2.3. LIODetect TB‐ST Serological Screening

Serum samples from camels and workers were screened with LIODetect TB‐ST (LIONEX Diagnostics and Therapeutics GmbH, Braunschweig, Germany) [21], a lateral‐flow immunochromatographic assay for antibodies to a panel of mycobacterial antigens. The assay was used as an exploratory screening tool. It has not been validated for estimating MTBC infection prevalence in dromedary camels, and camel‐specific sensitivity, specificity, likelihood ratios, and predictive values are unavailable. The rapid test evaluated in the 2007 UAE dromedary outbreak was a different Chembio lateral‐flow platform (later marketed as Camelid TB STAT‐PAK), not LIODetect TB‐ST [9]; those results therefore support the potential utility of antibody‐based screening in dromedaries but do not constitute validation of the assay used in the present study. Published performance estimates from cattle and human studies [14, 15] were treated only as contextual evidence and were not used to adjust the observed camel proportions. Throughout this manuscript, a valid test with a visible test band is termed LIODetect‐reactive or seroreactive; these terms describe antibody detection and do not denote confirmed MTBC infection, latent infection, or active tuberculosis.

**Figure 2:**
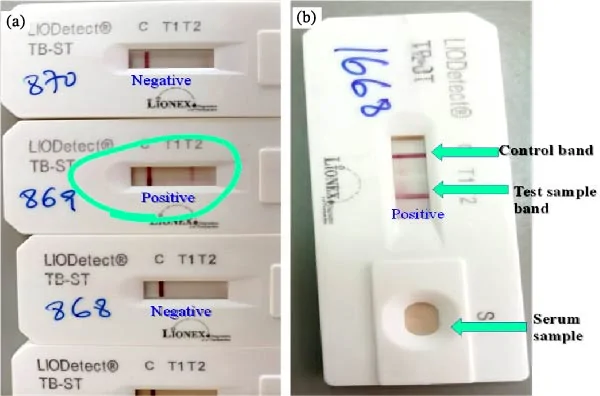
Representative LIODetect TB‐ST rapid‐test results and cassette interpretation. (a) A nonreactive result shows the control band only, whereas a reactive result shows the control band together with a visible test band at T1 and/or T2. (b) Enlarged reactive cassette showing the control band, test‐band positions, and sample well. In this study, any visible T1/T2 band, including a faint band, was classified as reactive when the control band was present. The labels visible on the original assay photographs reflect the contemporaneous positive/negative test notation; the manuscript uses reactive/nonreactive terminology because antibody detection does not independently establish MTBC infection or active tuberculosis.

Camel blood was collected into plain tubes, allowed to clot, and serum was separated. Following the study protocol and manufacturer instructions, 20 μL of serum was applied to the test pad, two drops of diluent were added, and the device was held at room temperature for 5 min. One additional drop of diluent was then added, and the result was read after a further 20 min. A visible control band was required for a valid result. The manufacturer grading scheme classified test‐band intensity as 0 (no visible band), 1 (faint), 2 (moderate), or 3 (strong); for the present binary analysis, any visible T1 and/or T2 test band (grade 1–3) together with a control band was classified as reactive, whereas a control band without a test band was classified as nonreactive (Figure 2). No separate equivocal category was used. Formal blinding of test readers to animal or worker metadata was not documented in the retained study records.

### 2.4. Postmortem and Histopathological Examination

Detailed postmortem examination was restricted to the 25 LIODetect‐reactive slaughtered camels. The 368 nonreactive slaughtered camels were not examined postmortem; therefore, lesions among nonreactive animals cannot be excluded. The pathological findings apply only to the seroreactive subset and cannot be used to estimate lesion prevalence in all slaughtered camels, false‐negative frequency, sensitivity, specificity, or positive predictive value. The parotid, mandibular, retropharyngeal, mediastinal, bronchial, and tracheobronchial lymph nodes and all seven lung lobes were examined. Hepatic and mesenteric lymph nodes were not consistently accessible because they were transferred immediately to another processing area. Gross lesions considered compatible with tuberculosis included firm, enlarged lymph nodes containing pale nodules and caseous material. Because granulomatous lesions are not pathognomonic for MTBC and may have other infectious causes, suspect tissues were evaluated by histopathology, Ziehl–Neelsen staining, and MTBC‐targeted PCR.

Suspect tissues were divided for histopathological and molecular examination. Samples for histopathology were fixed in 10% neutral‐buffered formalin, processed through graded alcohol and xylene, embedded in paraffin, and sectioned at 4 μm. Sections were stained with hematoxylin and eosin to characterize granulomatous lesions and with the Ziehl–Neelsen stain to detect acid‐fast bacilli. Mycobacterial culture was not performed, and the retained study records do not document whether this reflected biosafety, logistical, or specimen‐handling constraints.

### 2.5. IS6110 PCR

IS6110 PCR was performed only on lesion tissue from the two camels in which suspect retropharyngeal and mediastinal lymph‐node lesions were identified during postmortem examination. A 245‐bp region of IS6110 was amplified using primers INS1 (5′‐CGTGAGGGCATCGAGGTGGC‐3′) and INS2 (5′‐GCGTAGGCGTCGGTGACAAA‐3′) [22]. Each 20‐μL reaction contained 4.0 μL of 5× FIREPol Master Mix, 0.2 μL of each primer (10 μmol/L), 4 μL of the DNA template, and 11.6 μL of nuclease‐free water. Amplification consisted of initial denaturation at 95°C for 12 min, 30 cycles of 95°C for 30 s, 56°C for 60 s, and 72°C for 20 s; and final extension at 72°C for 5 min. Products were separated on 1.5% agarose gels stained with RedSafe. The detection of the expected IS6110 amplicon was interpreted as evidence of MTBC DNA in the sampled lesion, but IS6110 did not identify the infecting species or strain. Species‐specific PCR, spoligotyping, MIRU‐VNTR, whole‐genome sequencing, and culture were not performed.

### 2.6. Statistical Analysis

Data were audited against the retained individual‐level records before the analysis. The LIODetect‐reactive proportion was calculated as the number of reactive tests divided by the number tested and summarized with two‐sided exact Clopper–Pearson 95% confidence intervals. These proportions were not treated as estimates of true MTBC infection prevalence because camel‐specific assay sensitivity and specificity were unavailable. Unadjusted associations between categorical variables and seroreactivity were tested with the Fisher–Freeman–Halton exact test, an extension of Fisher’s exact test for contingency tables larger than 2 × 2. Exact testing was selected because several tables contained small or zero cell counts.

Firth bias‐reduced logistic regression, a penalized‐likelihood method that reduces small‐sample bias and yields finite estimates under complete or quasi‐complete separation, was used to estimate crude and adjusted odds ratios (ORs) with 95% profile‐likelihood confidence intervals [23, 24]. All recorded covariates considered epidemiologically relevant and available in each camel dataset were specified a priori; univariable *p*‐value screening was not used to select the multivariable models. The field‐camel model included region, holding type, age class, body condition, and origin; the single camel with an unknown origin was retained descriptively but excluded from regression. The slaughter‐camel model included sex, age group, and body condition. Global adjusted *p*‐values were obtained from penalized likelihood‐ratio tests. Model stability remained limited by the number of outcomes: the field model contained 32 reactive outcomes among 355 camels for eight slope parameters, and the slaughter model contained 25 reactive outcomes for five slope parameters. Firth regression mitigates separation and small‐sample bias but does not remove overfitting or unmeasured confounding; the estimates were therefore interpreted as exploratory. Holding‐level clustering could not be incorporated because unique holding identifiers were absent from the retained MTBC analytical dataset. No regression model was fitted for workers because only three reactive outcomes were observed.

## 3. Results

### 3.1. Seroreactivity Among Camels From Farms and Izbas

The 356 field camels came from 102 holdings distributed across Abu Dhabi, Eastern (Al Ain), and Al Dhafra regions, as described in Section 2.2.1. Thirty‐three camels were LIODetect‐reactive, giving a reactive proportion of 9.3% (exact 95% CI: 6.5%–12.8%). Reactivity was 5/78 (6.4%) in the Abu Dhabi Region, 19/168 (11.3%) in the Eastern Region, and 9/110 (8.2%) in Al Dhafra (Fisher–Freeman–Halton exact *p* = 0.466). By holding category, reactivity was 2/18 (11.1%) among camels sampled from commercial farms, 26/254 (10.2%) from random izbas, and 5/84 (6.0%) from regular izbas (exact *p* = 0.510).

No reactive results were detected among the 18 camels classified as young or the seven camels in poor body condition, but both categories were small. Exact tests showed no statistical evidence of heterogeneity in reactivity by age class, body condition, or recorded origin. The exploratory multivariable Firth model likewise did not identify a statistically supported association with region, holding type, age class, body condition, or origin; all category‐specific profile‐likelihood CIs included unity (Table 1). These animal‐level estimates do not account for within‐holding correlation because holding identifiers were unavailable in the retained MTBC dataset.

**Table 1:** LIODetect seroreactivity and exploratory association analysis among camels sampled from farms and izbas.

Variable	Category	Reactive/total	LIODetect reactive (%) (95% CI)	Crude OR (95% CI)	Adjusted OR (95% CI)	Exact *p* ^a^	Adjusted *p* ^b^
Region	Abu Dhabi	5/78	6.4 (2.1–14.3)	1.00 (reference)	1.00 (reference)	0.466	0.132
	Eastern (Al Ain)	19/168	11.3 (6.9–17.1)	1.74 (0.69–5.15)	1.37 (0.49–4.38)	—	—
	Al Dhafra	9/110	8.2 (3.8–15.0)	1.25 (0.43–4.00)	0.94 (0.29–3.30)	—	—
Holding type	Farm	2/18	11.1 (1.4–34.7)	2.19 (0.37–10.04)	1.86 (0.30–8.80)	0.510	0.245
	Random izba	26/254	10.2 (6.8–14.6)	1.68 (0.69–4.83)	1.56 (0.58–4.90)	—	—
	Regular izba	5/84	6.0 (2.0–13.3)	1.00 (reference)	1.00 (reference)	—	—
Age class	Young	0/18	0.0 (0.0–18.5)	0.25 (0.00–1.88)	0.21 (0.00–1.63)	0.393	0.122
	Adult	33/338	9.8 (6.8–13.4)	1.00 (reference)	1.00 (reference)	—	—
Body condition	Poor	0/7	0.0 (0.0–41.0)	0.64 (0.00–5.52)	0.76 (0.01–7.37)	0.914	0.607
	Medium	8/80	10.0 (4.4–18.8)	1.12 (0.47–2.46)	0.96 (0.38–2.16)	—	—
	Good	25/269	9.3 (6.1–13.4)	1.00 (reference)	1.00 (reference)	—	—
Origin	Born in UAE	30/330	9.1 (6.2–12.7)	1.00 (reference)	1.00 (reference)	1.000	0.398
	Introduced	2/25	8.0 (1.0–26.0)	1.05 (0.20–3.46)	1.01 (0.19–3.47)	—	—
	Unknown	1/1	100.0 (2.5–100.0)	—	—	—	—

Abbrevations: CI, confidence interval; MTBC, *Mycobacterium tuberculosis* complex; OR, odds ratio.

^a^Two‐sided Fisher–Freeman–Halton exact test; for 2 × 2 tables this is equivalent to Fisher’s exact test. Crude ORs were obtained from univariable Firth models as effect‐size estimates; univariable regression *p*‐values are not reported.

^b^Global penalized likelihood‐ratio test from the multivariable Firth model. Adjusted estimates are from a model including all listed variables. The single camel with unknown origin was reactive and was excluded from regression. No observations were missing for region, holding type, age class, or body condition. Holding identifiers were unavailable in the retained MTBC dataset, so within‐holding clustering could not be modeled.

### 3.2. Seroreactivity Among Slaughtered Camels

Among 393 slaughtered camels, 25 were LIODetect‐reactive, giving a reactive proportion of 6.4% (exact 95% CI: 4.2%–9.2%). Reactivity did not differ by sex (exact *p* = 0.641). Statistical heterogeneity was observed across age groups: 12/117 (10.3%) among camels aged <4 years, 4/158 (2.5%) among those aged 4–7 years, and 9/118 (7.6%) among those aged >7 years (exact *p* = 0.020). Reactivity also varied by body condition: 1/30 (3.3%) in poor, 0/63 (0%) in medium, and 24/300 (8.0%) in good condition (exact *p* = 0.028). These comparisons are exploratory and should not be interpreted as causal risk‐factor effects.

In the multivariable Firth model, global tests retained statistical evidence of heterogeneity by age group (*p* = 0.008) and body condition (*p* = 0.035). Camels aged 4–7 years had lower adjusted odds of reactivity than those aged >7 years (OR 0.37, 95% CI 0.11–1.13), although the category‐specific CI included unity. No reactive result occurred among the 63 camels with medium body condition; the bias‐reduced estimate was OR 0.11 (95% CI 0.001–0.79) relative to the good condition. Sparse cells, possible selection into the slaughter population, and unmeasured origin and management characteristics limit the biological interpretation (Table 2). All 25 reactive slaughtered camels underwent detailed postmortem examination; nonreactive slaughtered camels were not examined postmortem.

**Table 2:** LIODetect seroreactivity and exploratory association analysis among slaughtered camels at Al Bawadi Abattoir.

Variable	Category	Reactive/total	LIODetect reactive (%) (95% CI)	Crude OR (95% CI)	Adjusted OR (95% CI)	Exact *p* ^a^	Adjusted *p* ^b^
Sex	Female	20/295	6.8 (4.2–10.3)	1.00 (reference)	1.00 (reference)	0.641	0.077
	Male	5/98	5.1 (1.7–11.5)	0.79 (0.27–1.96)	0.52 (0.17–1.40)		
Age	<4 years	12/117	10.3 (5.4–17.2)	1.37 (0.57–3.40)	1.50 (0.58–3.95)	0.020	0.008
	4–7 years	4/158	2.5 (0.7–6.4)	0.34 (0.10–1.01)	0.37 (0.11–1.13)		
	>7 years	9/118	7.6 (3.5–14.0)	1.00 (reference)	1.00 (reference)		
Body condition	Poor	1/30	3.3 (0.1–17.2)	0.57 (0.06–2.35)	0.67 (0.07–2.89)	0.028	0.035
	Medium	0/63	0.0 (0.0–5.7)	0.09 (0.00–0.65)	0.11 (0.00–0.79)		
	Good	24/300	8.0 (5.2–11.7)	1.00 (reference)	1.00 (reference)		

Abbrevations: CI, confidence interval; MTBC, *Mycobacterium tuberculosis* complex; OR, odds ratio.

^a^Two‐sided Fisher–Freeman–Halton exact test; for 2 × 2 tables this is equivalent to Fisher’s exact test. Crude ORs were obtained from univariable Firth models as effect‐size estimates; univariable regression *p*‐values are not reported.

^b^Global penalized likelihood‐ratio test from the multivariable Firth model. Adjusted estimates are from a model containing sex, age group, and body condition. The medium‐body‐condition category contained no reactive camels; its estimate was obtained using bias‐reduced regression. No observations were missing for variables shown.

### 3.3. Pathological and Molecular Findings in Seroreactive Slaughtered Camels

**Figure 3:**
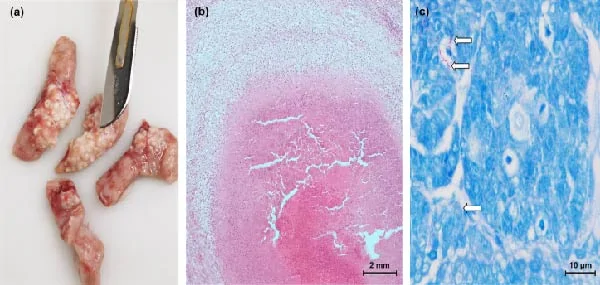
Gross and histopathological findings in lymph nodes from dromedary camels with TB‐compatible lesions. (a) Multiple pale nodules within an affected lymph node. (b) Granulomatous inflammation with central caseous necrosis in a hematoxylin‐and‐eosin‐stained section. (c) Ziehl–Neelsen‐stained section showing acid‐fast bacilli indicated by arrows. These features are compatible with tuberculous lymphadenitis but are not species‐specific; MTBC‐targeted molecular findings are presented in Figure 4. Scale bars are retained from the original micrographs.

Among the 25 LIODetect‐reactive slaughtered camels examined postmortem, two (8.0%) had lesions compatible with tuberculosis in the retropharyngeal and mediastinal lymph nodes. Because verification was restricted to reactive animals, 2/25 is a conditional descriptive proportion and is neither the prevalence of TB lesions among the 393 slaughtered camels nor the positive predictive value of LIODetect. Affected nodes were enlarged and firm, with multiple pale yellow‐white to gray nodules and caseous material on sectioning. Histological examination showed organized granulomatous inflammation with central caseous necrosis, epithelioid macrophages, occasional Langhans‐type multinucleated giant cells, peripheral lymphocytes, and fibrous encapsulation. Ziehl–Neelsen staining demonstrated slender red acid‐fast bacilli within granulomatous lesions and necrotic debris (Figure 3).

**Figure 4:**
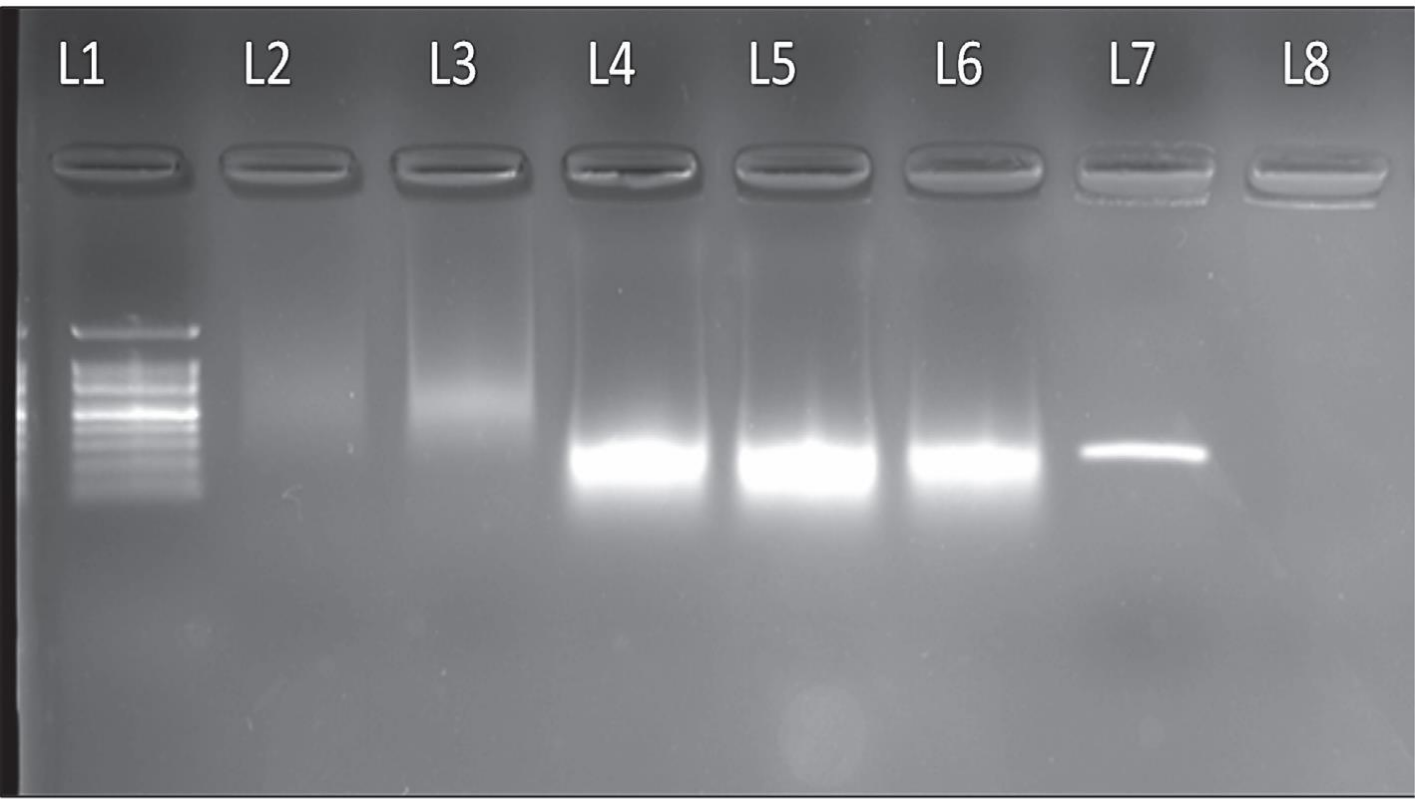
Agarose gel electrophoresis of IS6110 PCR products from suspect camel lymph‐node lesions. L1, molecular‐size marker; L2, negative control; L3–L6, lesion‐derived DNA preparations from the two affected camels; L7, *Mycobacterium bovis* positive control; L8, nontuberculous mycobacteria control. Bands at the expected 245‐bp amplicon size are visible in lesion‐derived preparations and in the positive control. PCR was interpreted at the animal level as detecting IS6110‐compatible MTBC DNA in lesion material from both affected camels; the assay did not determine MTBC species or strain.

IS6110 PCR was performed only on the lesion tissue from these two camels, and material from both animals yielded the expected 245‐bp amplicon. The concordant gross, histopathological, acid‐fast, and IS6110 findings strongly support MTBC‐associated tuberculous lesions in these animals while avoiding species‐level attribution. Acid‐fast staining alone is not specific for MTBC, and culture was not performed; however, detection of IS6110 in the same lesions provides MTBC‐targeted molecular support. Species‐level identification and strain characterization were not achieved, and the animals’ individual origins could not be confirmed.

### 3.4. Seroreactivity Among Abattoir Workers

Three of the 86 abattoir workers were LIODetect‐reactive, giving a reactive proportion of 3.5% (exact 95% CI: 0.7%–9.9%). Two reactive workers were aged >40 years, and one was aged ≤30 years; none of the 37 workers aged >30–40 years was reactive. All three reactive workers were butchers. Fisher–Freeman–Halton exact tests showed no statistical evidence of heterogeneity by age group (*p* = 0.105), job category (*p* = 1.000), employment duration (*p* = 0.612), or nationality group (*p* = 0.298) (Table 3). Only three reactive outcomes were observed, precluding reliable multivariable modeling. No clinical or microbiological confirmation of tuberculosis was available for these workers.

**Table 3:** LIODetect seroreactivity among abattoir workers according to recorded characteristics.

Variable	Category	Reactive/total	LIODetect reactive (%) (95% CI)	Exact *p* ^a^
Age	≤30 years	1/27	3.7 (0.1–19.0)	0.105
	>30–40 years	0/37	0.0 (0.0–9.5)	—
	>40 years	2/22	9.1 (1.1–29.2)	—
Job	Butcher	3/71	4.2 (0.9–11.9)	1.000
	Other	0/15	0.0 (0.0–21.8)	—
Duration	≤5 years	0/20	0.0 (0.0–16.8)	0.612
	>5–10 years	2/32	6.2 (0.8–20.8)	—
	>10 years	1/34	2.9 (0.1–15.3)	—
Nationality group	Bangladesh	0/20	0.0 (0.0–16.8)	0.298
	Egypt	0/11	0.0 (0.0–28.5)	—
	Pakistan	1/36	2.8 (0.1–14.5)	—
	Other African	1/7	14.3 (0.4–57.9)	—
	Other Asian	1/12	8.3 (0.2–38.5)	—

Abbrevations: CI, confidence interval; MTBC, *Mycobacterium tuberculosis* complex; OR, odds ratio.

^a^Two‐sided Fisher–Freeman–Halton exact test; for 2 × 2 tables this is equivalent to Fisher’s exact test. All participants were male. No observations were missing for the variables shown. Regression was not performed because only three reactive outcomes were observed. LIODetect reactivity in workers was not considered diagnostic of active or latent tuberculosis.

### 3.5. Comparison Across Study Populations

**Figure 5:**
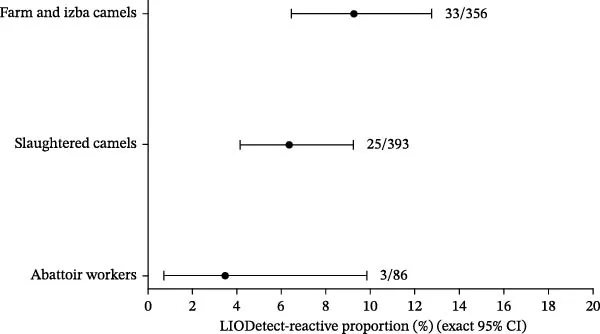
LIODetect‐reactive proportions in field camels, slaughtered camels, and abattoir workers. Points show observed reactive proportions; horizontal lines show exact two‐sided 95% Clopper–Pearson confidence intervals. Numerators and denominators are displayed beside each estimate. The estimates describe assay reactivity and should not be interpreted as true MTBC infection prevalence or as evidence of a transmission gradient.

The LIODetect‐reactive proportion was 9.3% in field camels, 6.4% in slaughtered camels, and 3.5% in abattoir workers (Figure 5). These are parallel assay‐reactivity estimates from different populations, not a transmission gradient. The animals and workers were not linked at the individual exposure or strain level, and no inference about the direction of transmission can be made from these comparisons.

## 4. Discussion

This study identifies a One Health surveillance signal across three components of the camel–abattoir–worker system in Abu Dhabi Emirate. LIODetect antibody reactivity occurred in field camels, slaughtered camels, and workers at Al Bawadi Abattoir, while two seroreactive slaughtered camels had TB‐compatible granulomatous lymphadenitis, acid‐fast bacilli, and IS6110‐detectable MTBC DNA in the lesion tissue. These findings are relevant to veterinary surveillance and meat inspection, but the study does not demonstrate camel‐to‐human transmission, estimate true MTBC prevalence, or identify the infecting MTBC species.

The interpretation of the serological component depends on the limitations of the assay. LIODetect TB‐ST has not been validated specifically in dromedary camels, and no camel‐specific sensitivity, specificity, likelihood ratios, or predictive values are available. The most directly relevant UAE evidence comes from the 2007 racing‐herd outbreak, in which a different Chembio lateral‐flow rapid test detected antibodies in all three dromedaries with culture‐confirmed *Mycobacterium bovis* infection and was nonreactive in the remaining 55 animals [9]. That study was small, used a different assay, and verified infection postmortem only in seroreactive animals; it therefore demonstrates potential diagnostic utility rather than formal sensitivity or specificity for LIODetect TB‐ST. Other camel studies using rapid antibody formats also provide supportive but assay‐specific evidence [12, 13]. In cattle, the related LIONEX Animal TB rapid test had 54% sensitivity and 98.8% specificity against a combined SICTT/IFN‐γ reference and substantial agreement with SICTT (*κ* = 0.63) [14]. In a human evaluation, LIODetect TB‐ST had 73.3% sensitivity and 97.4% specificity [15]. These values cannot be assumed for dromedaries because humoral responses, disease stage, antigen recognition, prior sensitization, and test spectrum differ across hosts. The 2007 UAE study also found stronger avian than bovine PPD responses in some presumed uninfected camels, which the authors considered compatible with exposure to environmental mycobacteria [9]. Although that observation concerns tuberculin testing rather than LIODetect, it illustrates the broader diagnostic complexity of mycobacterial exposure in camelids. WOAH guidance likewise notes that antibody responses to MTBC often emerge later than cell‐mediated responses and that serological tests are generally less sensitive than cell‐mediated assays, although they may have complementary value in selected animal settings [16]. Potential antibody cross‐reactivity with environmental or nontuberculous mycobacteria cannot be excluded in this study. For these reasons, the reported percentages are LIODetect‐reactive proportions, not prevalence estimates of MTBC infection.

The 9.3% reactive proportion among field camels indicates that antibody reactivity was detectable across sampled holdings, but its epidemiological meaning remains uncertain. The parent field survey used multistage random sampling across 102 holdings, which strengthens geographic coverage [17]; however, unique holding identifiers were not retained in the MTBC analytical dataset. Consequently, within‐holding correlation could not be modeled, and herd‐level determinants could not be assessed. The available TB dataset also lacked herd size, mixed‐species contact, detailed movement history, clinical history, milk‐management practices, and biosecurity measures. These limitations restrict inference from the absence of statistically supported animal‐level associations.

Among slaughtered camels, global tests indicated heterogeneity in seroreactivity by age group and body condition. These results should be viewed as exploratory signals rather than biological risk factors. The age pattern was nonmonotonic, no reactive tests occurred in the medium‐body‐condition category, and several estimates had wide confidence intervals. Selection into the slaughter population, sparse cells, unmeasured origin and management, and possible age or condition misclassification could generate or amplify these patterns. Replication in prospectively sampled slaughter populations with systematic covariate collection is needed before these variables are used for risk‐based control.

The pathological and molecular findings in the two lesion‐bearing camels are more specific than the antibody results. Both animals had caseating granulomatous lymphadenitis with acid‐fast bacilli, and IS6110 DNA was detected in the same lesion material. Similar pathological presentations have been reported in dromedaries in several countries [5, 6, 25, 26]. Nevertheless, granulomatous lesions and acid‐fast organisms have differential diagnoses, including nontuberculous mycobacteria, and IS6110 establishes MTBC‐targeted DNA detection rather than species or strain identity. Culture was not performed, and the reason was not documented in the retained records. Region‐of‐difference PCR, culture, spoligotyping, MIRU‐VNTR, or whole‐genome sequencing would be required to distinguish *M. bovis*, *M. tuberculosis*, *M. caprae*, or another MTBC member and to investigate the epidemiological linkage.

Selective postmortem verification is one of the principal limitations of the study. Only the 25 LIODetect‐reactive slaughtered camels underwent detailed examination, while all 368 nonreactive camels remained unverified. Important lesions could therefore have been present among non reactive animals, particularly if antibody sensitivity in dromedaries is incomplete. This partial verification bias prevents estimation of lesion prevalence, false‐negative frequency, sensitivity, specificity, or positive predictive value. The 2/25 figure should be read only as the proportion of examined seroreactive camels in which TB‐compatible lesions were identified, not as a diagnostic‐performance measure.

The worker component identified three reactive individuals, all butchers, but antibody reactivity cannot diagnose active or latent tuberculosis. Clinical assessment, chest imaging, interferon‐γ release testing, sputum examination, culture, and molecular confirmation were not part of the available study data, and referral outcomes were not documented. Detailed exposure intensity, personal protective equipment use, previous TB history, BCG vaccination, and household exposure were also unavailable. The human findings should therefore be treated as an occupational‐health surveillance signal that supports appropriate clinical referral and workplace risk assessment, not as evidence of occupational MTBC infection or zoonotic transmission [27, 28].

A balanced One Health interpretation must allow for several possible sources of MTBC exposure. Human‐adapted *M. tuberculosis* can infect livestock in settings of close animal–human contact [29–31], while animal‐adapted MTBC members can infect people [3, 4]. This study cannot determine whether the camel findings arose from other camels, other livestock, humans, or animal movement because camel and worker isolates were not available for comparative typing. Al Bawadi Abattoir nevertheless illustrates the value of slaughter facilities as sentinel surveillance points: animals from multiple sources converge in one location, where systematic meat inspection, targeted sampling, and traceable movement records can detect infection signals that may be missed on farms. Integrating abattoir surveillance with occupational‐health procedures would strengthen early detection without presuming a direction of transmission.

Several constraints should frame interpretation: the study covered one emirate and one abattoir; the MTBC dataset did not retain holding identifiers; the LIODetect assay lacks dromedary‐specific validation; formal laboratory blinding was not documented; seasonal effects were not analyzed; nonreactive slaughtered camels were not examined postmortem; culture and species‐level molecular characterization were not performed; slaughter‐camel origins were incomplete; and worker clinical follow‐up and detailed exposure histories were unavailable. Future surveillance should use stratified multistage sampling with preserved herd identifiers, systematically examine slaughtered animals irrespective of serological status, validate camel diagnostic algorithms against culture and molecular reference standards, and characterize isolates using species‐specific PCR and preferably whole‐genome sequencing. Linking animal movements, abattoir findings, and occupational‐health evaluation would provide the evidence needed to assess transboundary introduction, local transmission pathways, and practical control options [3, 32].

## 5. Conclusion

LIODetect antibody reactivity was detected in field camels, slaughtered camels, and abattoir workers in Abu Dhabi Emirate. These results describe seroreactivity and should not be interpreted as true MTBC infection prevalence, active disease, or a transmission gradient. Among the 25 seroreactive slaughtered camels examined postmortem, two had TB‐compatible granulomatous lymphadenitis with acid‐fast bacilli and IS6110‐detectable MTBC DNA. Species‐level identification was not achieved, and nonreactive slaughtered camels were not examined, preventing the estimation of lesion prevalence or serological diagnostic accuracy. The study does not demonstrate camel‐to‐human transmission. Its principal contribution is to identify a surveillance signal and specific design priorities for future work: validated camel diagnostics, systematic postmortem examination irrespective of serostatus, culture and genomic characterization, traceable animal movements, preserved herd identifiers, and linked veterinary and occupational‐health surveillance.

## Author Contributions

Gobena Ameni conceived the study, led the analysis, and drafted the original manuscript. Aboma Zewude and Temesgen Mohammed collected field data and conducted serological and PCR procedures. Asha Antony and Robert Barigye conducted and interpreted the pathological examinations. Tsegaye Shamebo, Nabeeha Hassan Abdelgaleel, Mohamud Sheek‐Hussein, Balázs Ádám, and Markos Tibbo critically reviewed and edited the manuscript.

## Funding

This work was supported by the United Arab Emirates University UPAR Funding Program (Grant 12F033).

## Disclosure

All authors reviewed and approved the final manuscript and accept responsibility for the integrity of the work. The authors reviewed and edited all outputs, verified the cited sources and numerical results, and take full responsibility for the accuracy, originality, and integrity of the manuscript.

## Ethics Statement

The animal study was authorized by the Ministry of Climate Change and Environment of the United Arab Emirates on February 8, 2022 (Ref. MOCCEA/EA/2022). The animal and human components of the study were approved by the Abu Dhabi Health Research and Technology Ethics Committee (Ref. DOH/CVDC/2022/1136). The human component was conducted in accordance with the Declaration of Helsinki and applicable national and institutional requirements. Written informed consent was obtained from all participating abattoir workers. Animal sampling, handling, and postmortem procedures were conducted in accordance with applicable institutional and national animal‐welfare requirements.

## Conflicts of Interest

The authors declare no conflicts of interest.

## Data Availability

Deidentified data and reproducible statistical code supporting the findings are available from the corresponding author upon reasonable request, subject to the conditions of the ethics approvals and institutional data‐governance requirements.
